# Editorial: Structural Valve Degeneration and Failure in Transcatheter and Surgical Bioprosthesis

**DOI:** 10.3389/fcvm.2020.00058

**Published:** 2020-04-21

**Authors:** Chiara Fraccaro, Darren Mylotte, Massimo Napodano

**Affiliations:** ^1^Interventional Cardiology Unit, Department of Cardiac, Thoracic, Vascular Sciences and Public Health, University Hospital of Padua, Padua, Italy; ^2^Department of Cardiology, Galway University Hospital, SAOLTA Health Care Group, and National University of Ireland, Galway, Ireland

**Keywords:** structural valve degeneration (SVD), transcatheter heart valve (THV), bioprosthesis, valve dysfunction, bioprosthesis failure, transcatheter heart valve failure

With the increasing prevalence of valvular heart disease worldwide, the need for heart valve replacements is expected to hit as well. To date, the majority of them are performed with biological heart valves (BHV), which can be implanted either surgically or by a micro-invasive transcatheter approach. Despite the numerous advantages of BHV, they are not as mechanically robust compared to mechanical heart valves and exhibit limited durability in particular in younger patients. In recent times, the indications for transcatheter aortic valve replacement (TAVR) progressively extended also to lower risk and younger patients, with longer life expectancy.

Therefore, many concerns and discussions are grown up among cardiological and surgical community, regarding the durability of transcatheter prostheses in comparison to surgical implanted ones. In this edition, we cover broad aspects related to pathological mechanisms, classification, and treatment of structural valve degeneration (SVD).

The article by Li describes the multifaceted process of age-dependent SVD, exploring also emergent immunologic insights into this phenomenon such as antibody reactivity to different xenogeneic glycans. The author analyzes advantages and drawbacks of the different anti-calcification processing technologies and also provides an overview of the novel engineering valve designs utilizing Gal-free animal tissues which would be unaffected by anti-Gal antibody-mediated injury. Transcatheter heart valves, continuing to employ dead (non-vital) tissue with the basic technology of chemical fixation of bovine or porcine tissues, are still subject to SVD. However, the possibility for comparison between surgical and transcatheter bioprosthesis durability across studies is limited not only by the different period of application of the two methods (being SAVR performed for a longer time than TAVR), but also by the lack of a consensus definition for SVD. The article by Sawaya et al. is a critical review of the standardized criteria to define SVD and valve failure of both transcatheter and surgical bioprosthesis, recently introduced by the European Association of Percutaneous Cardiovascular Interventions (EAPCI), the European Society of Cardiology (ESC), and the European Association for Cardio-Thoracic Surgery (EACTS), that aim at generating uniformity in data reporting in future studies assessing the long-term durability of surgical and transcatheter bioprosthesis.

Transcatheter valve implantation technique is also frequently adopted as preferred therapeutic option for the treatment of surgically implanted degenerated bioprosthesis as well (valve-in-valve procedure), being surgical re-do at higher risk in most of the cases. The case reported by Ristalli et al. demonstrates how widely this technique has been developing, being used in different anatomical location and also in very critical clinical settings. However, valve-in-valve procedure can present some pitfalls and must be carefully planned. The most critical issue is probably the risk of coronary occlusion. The two review articles by Bernardi et al. and by Valvo et al. discuss the importance of pre-procedural assessment with multimodality imaging, in order to choice the best therapeutic option according to anatomical findings and to prevent coronary occlusion by means of dedicated interventional strategies.

The remarkable progress of interventional cardiology in the field of structural valve interventions has led to a substantial increase in the number of patients treated with transcatheter heart valve.

We believe that the topic of structural valve degeneration and failure in transcatheter and surgical bioprosthesis offers the opportunity for readers to know the challenges and potential future directions of interventional cardiology in structural valve disease today, and we hope that more researchers and physicians will appreciate this important and interesting, but still developing field of interventional cardiology.

## Author Contributions

CF has prepared the editorial with the consent from MN and DM.

## Conflict of Interest

The authors declare that the research was conducted in the absence of any commercial or financial relationships that could be construed as a potential conflict of interest.

